# An infant with 49XXXXY syndrome: a case report

**DOI:** 10.1186/s13256-021-03188-4

**Published:** 2021-12-30

**Authors:** N. P. G. C. R. Naotunna, C. Liyanage, N. Atapattu

**Affiliations:** 1 Endocrinology and Diabetic Unit, Lady Ridgeway Hospital, Colombo, Sri Lanka; 2 Paediatric Unit, Provincial General Hospital, Badulla, Sri Lanka

**Keywords:** 49XXXXY, Ambiguous genitalia, Dysmorphism

## Abstract

**Background:**

49XXXXY syndrome is the rarest X chromosome aneuploidy, with approximate incidence of 1:85,000–100,000 male births. Worldwide, around 100 cases have been reported. In this report, we describe one such case seen in Sri Lanka.

**Case presentation:**

A 10-day-old Sri Lankan neonate born in a tertiary care center was referred to the pediatric endocrinology unit of Lady Ridgeway Hospital due to detection of ambiguous genitalia at birth. He was the first child born to nonconsanguineous healthy parents following an uncomplicated antenatal period. He was born at term via normal vaginal delivery, with a birth weight of 2.385 kg. The baby was active, and there was no documented hypoglycemia or alteration in basic biochemical investigations. On examination, the child had hypertelorism, upslanting palpebral fissures, flat occiput, and mild webbing of the neck. System examination was normal. Genitalia examination revealed bifid scrotum, perineal urethra, 2 cm phallus, and bilateral testis *in situ*. Hormonal analysis, including dehydroepiandrosterone sulfate, testosterone, and 17-OH progesterone levels, was normal except for an elevated level of follicle-stimulating hormone, indicating gonadal dysgenesis. Ultrasound of the abdomen detected testis located at bilateral inguinal canal, and no Müllerian structures were visible. Echocardiography showed a small patent foramen ovale with otherwise normal heart. Chromosome analysis revealed 49XXXXY syndrome.

**Conclusion:**

49XXXXY syndrome should be entertained as a rare possibility for ambiguous genitalia, and karyotyping is an essential investigation for evaluation of such patients.

## Background

49XXXXY syndrome is a rare chromosomal aneuploidy, with a few hundred case reports published all over the world [2]. The classic triad of symptoms includes mental retardation, hypogonadism, and radioulnar synostosis with several other congenital malformations, associated medical conditions, and psychological impacts. Though many associations have been described as single case reports, to date genital malformations and ambiguity are not well described in literature. This case report aims to spotlight the association of genital ambiguity and 49XXXXY syndrome.

## Case presentation

A 10-day-old Sri Lankan Tamil neonate born in a tertiary care center was referred to Lady Ridgeway Hospital for further evaluation of ambiguous genitalia at birth. He was the first child born to nonconsanguineous healthy young parents following uncomplicated antenatal and perinatal periods at term via normal vaginal delivery, with a birth weight of 2.385 kg.

Neonatal examination revealed bifid scrotum, perineal urethra, 2 cm phallus, and palpable bilateral small testis. In addition, he had some dysmorphic features such as hypertelorism, upslanting palpebral fissures, flat occiput, and mild webbing of the neck. His length and occipital frontal circumference (OFC) at birth were 47 and 32 cm, respectively. During the hospital stay, the baby was well fed and his serum electrolyte levels and serum calcium levels remained normal throughout. Moreover, there were no documented hypoglycemic events or neonatal jaundice.

Subsequent investigations on day 14 of life showed normal levels of dehydroepiandrosterone (DHEA), testosterone, and 17-OH progesterone (17-OHP), with elevated serum follicle-stimulating hormone level suggesting gonadal failure (Table 1). Ultrasound of abdomen and scrotum revealed normal adrenal glands with no evidence of persistent Müllerian structures. There was no radiological evidence of radio ulnar synostosis. Karyotyping demonstrated chromosomal aneuploidy of 49XXXXY syndrome. Parents were advised to register the sex of the child as male and advised regarding follow-up and the need for hormone replacement therapy during puberty.

**Table 1:** Investigation summary

Investigation	Value	Reference range
Serum sodium	143 mmol/L	135–150 mmol/L
Serum potassium	4.7 mmol/L	3.5–5.3 mmol/L
FSH	22.59 IU/L	0.09–2.41 IU/L
LH	12.69 IU/L	0.19–3.81 IU/L
DHEAS	1.269 nmol/L	0.9–5.8 nmol/l
Testosterone	6.3 nmol/L	2.6–13.9 nmol/l
17-OHP, 0 minute	8.6 nmol/L	
17-OHP, 30 minute	18.9 nmol/L	<30 nmol/L

*FSH* follicle-stimulating hormone; *LH* luteinizing hormone; *DHEAS* dehydroepiandrosterone sulfate; *17-OHP* 17-hydroxyprogesterone

The child was followed up at the endocrinology clinic at 3 months to assess growth and development. Surgical referral was done at 6 months of age for correction of perineal hypospadias, and surgery is planned for 1 year and 3 months of age.

At age 1 year, the child’s growth parameters included weight below the 3rd centile (6.8 kg) and length between the 3rd and 15th centile (72 cm) according to WHO growth charts. His development at the age of 1 year was delayed, and he was commenced on physiotherapy, occupation therapy, and speech therapy at the local hospital. Follow-up ultrasound (USS) of abdomen and pelvis at 1 year was normal except for nonobstructive right-side renal calculus of 3.5 mm.

## Discussion

In 1960, Fraccaro *et al*. reported the first case of 49XXXXY syndrome [1]. This is the rarest X chromosome aneuploidy, with approximate incidence of 1:85,000–100,000 male births [2]. Nondisjunction of X chromosomes during both meiosis I and II is the probable chromosomal anomaly leading to 49XXXXY syndrome [3, 4].

Initially this was considered as a variant of Klinefelter syndrome, but the prevalence of moderate to severe mental retardation with low intelligence quotient (IQ) and multiple associated malformations in 49XXXXY syndrome demarcates the differences between these two clinical entities [2].

The classic triad of symptoms includes mental retardation, hypogonadism, and radioulnar synostosis, though our patient did not have the latter finding [2]. Other clinical manifestations include microcephaly with short stature; distinct facial features such as round face, ocular hypertelorism, upslanting palpebral fissures, flat nasal bridge, and cleft palate; musculoskeletal defects such as genu valgum, pes cavus or planus, clinodactyly, scoliosis, hip dysplasia and hypotonia; congenital heart defects, with patent ductus arteriosus being the most common [5]; and delayed neurocognitive development with low IQ levels. Moreover, it can be associated with immunodeficiency, making patients susceptible to infections.

A few case reports have highlighted the association of genital ambiguity with this syndrome [7, 8]. One such case describes a 2-month-old baby with micropenis and right undescended testes. Another newborn had a normal stretched penile length with penoscrotal hypospadias and bifid scrotum, with 1 ml testes *in situ* bilaterally. A 26-month-old baby had bilateral undescended testes with the penis buried with a urethral shelf at the urethral opening and penoscrotal webbing. In contrast to these, our patient had a unique presentation with micropenis, perineal hypospadias, and bifid scrotum with small palpable testes.

There has been a reported case of diabetes mellitus in an 18-year-old 49XXXXY patient, though this is common among children with Klinefelter syndrome [9].

Behavioral and development aspects in view of 49XXXXY have been studied widely, and many elderly patients with this syndrome had emotional lability, low frustration levels and shyness, occasional irritability, temper tantrums, and resistance to routine change [2, 6]. Marked delay was noted in language and speech development, with a clear discrepancy between language expression and comprehension. This child already had speech delay, and he needed further follow-up for assessment.

Hypergonadotropic hypogonadism needs to be evaluate at the age of puberty, and hormone replacement therapy with intramuscular (IM) testosterone should be initiated under endocrinology follow-up.

## Conclusion

Knowing the association of 49XXXXY syndrome in this child with ambiguous genitalia is beneficial as a multidisciplinary approach can be adopted for further management to ensure quality of life. Also, it is important to consider this rare possibility in a child with ambiguous genitalia and to stress the importance of karyotyping for evaluation.

## Data Availability

Not applicable.
